# Methods for detecting highly pathogenic avian influenza H5N1 virus in dairy processing environments

**DOI:** 10.3389/fmicb.2026.1842786

**Published:** 2026-06-04

**Authors:** Pablo Puchades-Colera, Inés Girón-Guzmán, Alba Pérez-Cataluña, Gloria Sánchez

**Affiliations:** VISAFELab, Department of Preservation and Food Safety Technologies, Institute of Agrochemistry and Food Technology, IATA-CSIC, Paterna, Valencia, Spain

**Keywords:** capsid integrity, fomites, HPAI H5N1, influenza A virus, milk

## Abstract

The spread of highly pathogenic avian influenza (HPAI) H5N1 into mammals, including U.S. dairy cattle, underscores the need for effective surveillance in milk and environmental samples. This study evaluated molecular methods to detect influenza A virus (IAV) in various milk types and dairy farm surfaces. An aluminum chloride (AlCl_3_) precipitation method was optimized for virus concentration in milk, combined with magnetic bead-based nucleic acid extraction and Plant RNA Isolation Aid. Mean recoveries of inactivated HPAI H5N1 were 22.46 ± 11.86% and 17.22 ± 6.15% in pasteurized and raw milk, respectively. Detection limits (LoD_95%_) for H3N2 were 2.9 × 10^5^ genome copies (gc)/L (raw) and 8.13 × 10^4^ gc/L (pasteurized); for H5N1, 1.20 × 10^4^ gc/L (raw) and 3.10 × 10^5^ gc/L (pasteurized). Capsid integrity RT-qPCR using propidium monoazide (PMAxx™), platinum (IV) chloride (PtCl_4_), and *Crosslinker* (CL), showed PtCl_4_ best eliminated signals from heat-inactivated virus, but only after extreme heat (99 °C, 5 min). For surfaces, ISO 15216:1-based swabbing with PBS outperformed sponge-based methods, especially on stainless steel and silicone. Virus recovery varied by surface and matrix. These results support standardized sampling and concentration protocols for HPAI H5N1 monitoring in dairy environments.

## 1 Introduction

As of April 2026, the highly pathogenic avian influenza (HPAI) H5N1 virus continues to drive a widespread global panzootic in birds and mammals. First detected in domestic dairy cattle in late March 2024, the B3.13 genotype of HPAI H5N1 has since spread across multiple U.S. states, resulting in at least 71 confirmed human cases and 2 fatality (CDC, 2025). Most infections have occurred among individuals with direct contact with infected livestock, poultry and other animals or unknown exposure sources (CDC, 2025). Recently, contact with or consumption of raw milk from affected cows has been identified as a potential source of human infection (Garg et al., 2025). In early 2025, a new genotype of the HPAI H5N1 strain, known as genotype D1.1, was detected in dairy cattle in Nevada (USDA, 2025). This marks the first detection of this genotype in cows, as previous cases were associated with the B3.13 genotype. The emergence of the D1.1 and D1.3 strains has raised concerns due to its potential for wider spread and its association with severe human infections, including fatalities (Rolfes et al., 2025; Zhang et al., 2025).

Infected cattle have exhibited symptoms such as reduced appetite, decreased milk production, and abnormal milk appearance, including thickening and discoloration (Kamel et al., 2025). Lactating cows appear to be particularly affected; however, clinical signs have been observed in fewer than 10% of cows within affected herds (Burrough et al., 2024; Kamel et al., 2025). Research has shown that the HPAI H5N1 virus can replicate in the mammary gland epithelial cells of dairy cows, leading to high viral titers, up to 10^8.8^ 50 % tissue culture infectious dose (TCID_50_)/mL, in milk of infected cows (Caserta et al., 2024; Le Sage et al., 2024; Nelli et al., 2024). The detection of the virus in unpasteurized milk from infected cows raises concerns about potential transmission through dairy products (Burrough et al., 2024; Guan et al., 2024). While pasteurization inactivates the virus (Alkie et al., 2025; Kamel et al., 2025; Schafers et al., 2025), health authorities continue to assess the risks associated with raw milk consumption. Furthermore, the presence of HPAI H5N1 RNA has been found on surfaces within affected farms (Butt et al., 2024; Halwe et al., 2024; Kamel et al., 2025; Le Sage et al., 2024; Marshall et al., 2024; Stachler et al., 2025), suggesting environmental contamination that may contribute to transmission among animals and potentially to humans (Bellido-Martín et al., 2026; Butt et al., 2024; Kaiser et al., 2025; Le Sage et al., 2024; Wood et al., 2010).

To address the outbreak, the U.S. Department of Agriculture (USDA), in collaboration with state agencies, has implemented measures to monitor and control the outbreak. These efforts include bulk milk testing to track viral circulation in dairy farms, enhanced testing protocols, genome sequencing of the virus, and epidemiological investigations to understand transmission pathways (Alvarez et al., 2025; Gontu et al., 2025; Stenkamp-Strahm et al., in press). Surveillance has also been expanded to include dairy silos and nearby wildlife populations to better assess viral spread. While the Centers for Disease Control and Prevention (CDC) currently assesses the public health risk to the general population as low, individuals with occupational exposure to livestock are urged to follow strict biosecurity and personal protective measures. Continued surveillance, diagnostic innovation, and research into viral infectivity will be essential to contain the spread of HPAI H5N1 and protect both animal and human health (Bartlett et al., 2025; Kamel et al., 2025; Lail et al., 2025; Martin et al., 2024). Given the detection of viral RNA in milk and on farm surfaces, there is an urgent need for rapid and reliable detection methods to enhance surveillance and enable timely response measures.

Several studies have assessed procedures for extracting viral nucleic acids from milk (Sayed et al., 2020; Snoeck et al., 2024; Stachler et al., 2025; Suarez et al., 2025; Zulli et al., 2024). However, to our knowledge, none have specifically examined methods for concentrating IAV in milk, a potentially crucial step when viral loads are low or genomes could be damaged by technological treatments, as in bulk tank milk. In this context, although HPAI H5N1 can be shed in high concentrations in milk, dairy matrices are not currently included in the ISO 15216-1:2017 method for virus detection in food. No standardized procedures are yet available for these matrices, and previous studies have often applied protocols originally designed for shellfish (Hennechart-Collette et al., 2022). Therefore, concentration methods must be re-evaluated and optimized to account for new viral pathogens and contamination levels. By validating such methods, we contribute to the establishment of robust tools for virus detection in milk and support the development of large-scale surveillance programs.

Furthermore, environmental contamination poses an additional occupational exposure risk for both animals and dairy workers, as HPAI H5N1 can persist in the farm environment (Le Sage et al., 2024; Kaiser et al., 2025). Implementing standardized surface-testing methods for HPAI H5N1 on dairy farms is therefore crucial for early detection and containment, helping to prevent viral spread, ensure milk safety, and protect both animal and human health within the dairy industry. These approaches could also be adapted for surface surveillance in the poultry sector.

Ongoing research is also focused on assessing the infectivity of viruses detected in milk and the farm environment, an essential step in evaluating transmission risks to both animals and humans. Conventional RT-qPCR assays cannot differentiate between infectious and non-infectious IAV particles, while infectivity is traditionally confirmed through inoculation in embryonated chicken eggs or in Madin–Darby canine kidney (MDCK) cells (Guan et al., 2024; Spackman et al., 2024a,b). The integration of intercalating markers into RT-qPCR protocols addresses this limitation by selectively amplifying genomes from intact, potentially infectious viruses. This approach has demonstrated high efficacy for IAV and coronaviruses (Canh et al., 2022) and may provide a rapid, reliable screening method to assess viral infectivity in milk and dairy products, offering valuable insights for both the dairy industry and public health surveillance.

Therefore, this study aimed to evaluate and optimize an aluminum-based virus concentration method for the detection of influenza A virus in milk, using the IAV H3N2 strain and an inactivated HPAI H5N1 strain. Additionally, porcine epidemic diarrhea virus (PEDV) was used as an enveloped virus model and mengovirus (MgV) as a non-enveloped model virus and process controls, in accordance with ISO 15216-1:2017 standards. The study also assessed the performance of capsid integrity RT-qPCR pretreatments and compared surface sampling methods for effective virus monitoring in dairy environments.

## 2 Materials and methods

### 2.1 Viruses, cell lines and milk samples

Influenza A virus (IAV) H3N2 (ATCC VR-1680) was propagated in MDCK cell line (ATCC CCL-34) according to ATCC recommendations. Infectious viruses were enumerated by determining the TCID_50_ in 96-well microtiter plates with eight wells per dilution and 20 μL of inoculum per well using the Spearman-Karber method (Dougherty, 1964; Falcó et al., 2018). The inactivated HPAI H5N1 virus A/Larus ridibundus/Spain/CR4063/2023 (kindly provided by Prof. Natàlia Majó, IRTA, CReSA) was prepared by heat inactivation in a thermoblock at 70 °C for 1 h.

The semipurified stock of PEDV strain CV777 was kindly provided by Dr. Carvajal (Department of Animal Health, Faculty of Veterinary Medicine, University of León). The MgV vMC0 (CECT 1,00,000) stock was obtained as described by Falcó et al. (2018).

Ultra-high temperature (UHT) milk and pasteurized milk were purchased from a local store. Fresh raw milk was obtained from a national farm, transported under refrigeration, and stored at 4 °C until use. All samples were immediately refrigerated upon arrival.

### 2.2 Viral detection and quantification

Viral detection for the analyzed viruses (i.e., IAV, PEDV, MgV) was performed by RT-qPCR with primers and probes described previously (Supplementary Table S1), using the One Step PrimeScript™ RT-PCR Kit (Perfect Real Time) (Takara Bio). Detection of IAV was performed using three RT-qPCR assays: a generic RT-qPCR for IAV detection targeting matrix (M) gene (Assay A, CDC, 2021); a specific RT-qPCR to detect H5Nx subtypes (Assay B, Wolfe et al., 2024); and one specific to detect H5 clade 2 (Assay C, WHO, 2021). Detailed information on the primers, probes, amplification conditions, and the limits of detection and quantification for each virus used in this study is provided in Supplementary Table S1. All RT-qPCR assays were performed in duplicate using the QuantStudio™ 5 Real-Time PCR (Applied Biosystems). To assess the presence of PCR inhibitors, 10-fold dilutions of the extracted nucleic acids were tested in duplicate for all targeted viruses. Standard curves were generated using commercially available Twist Synthetic Influenza H1N1 RNA control (Twist Bioscience, San Francisco, USA) for Assay A; purified HPAI H5N1 RNA (provided by CReSA) for assays B and C; and purified RNA from PEDV and MgV for quantification of these viral controls. These RNAs were used as positive controls, while negative control consisted in nuclease-free water.

### 2.3 Method comparison in milk samples

A rapid method for milk concentration was evaluated using the aluminum-based adsorption-precipitation (AlCl_3_) method, which has been previously applied for virus concentration in water samples (AAVV, 2018; Girón-Guzmán et al., 2024; Randazzo et al., 2020). To evaluate the performance of the method, 200 mL of each type of milk was inoculated with IAV H3N2, inactivated HPAI H5N1, PEDV (as a model for enveloped viruses), and MgV (as a model for non-enveloped viruses), and subsequently concentrated using the AlCl_3_ method. Briefly, 200 mL of milk samples were transferred into 250 mL centrifuge bottles (ThermoFisher Scientific, Rochester, USA) and the pH was adjusted to 6.0 using 0.1 N HCl and monitored with pH strips (ThermoFisher Scientific). Precipitation with Al(OH)_3_ was obtained by adding 1:100 parts 0.9 N AlCl_3_ (Acros Organics, Geel, Belgium). The pH was further readjusted to 6.0, and the sample was mixed at 150 rpm for 15 min at room temperature (RT) on an orbital shaker. Subsequently, the sample was centrifuged at 1,700 × *g* for 20 min, and the resulting pellet was resuspended in 10 mL of 3 % beef extract (pH 7.4) (Condalab, Madrid, Spain) and transferred to 50 mL centrifuge tubes. The pellet was then mixed at 150 rpm for 10 min, followed by centrifugation at 1,900 × *g* for 30 min at RT. The obtained pellet was resuspended in 1 mL of phosphate buffer solution (PBS) and reserved before nucleic acid extraction using the magnetic bead-based method. All experiments were performed in triplicate.

In parallel with the concentration step, our study compared the analytical performance of the Maxwell^®^ RSC Instrument (Promega, Madison, USA) nucleic acid extraction protocol using the Maxwell RSC Pure Food GMO and authentication kit (Promega) and the “Maxwell RSC Viral Total Nucleic Acid” running program, with or without the addition of 40 μL Plant RNA Isolation Aid (Thermofisher Scientific) as a pretreatment to remove inhibitors naturally present in dairy samples (Snoeck et al., 2024). A recovery control consisting of 300 μL of PBS spiked with IAV H3N2, inactivated HPAI H5N1, PEDV and MgV was used to monitor mean recovery rates.

### 2.4 IAV H3N2 and HPAI H5N1 detection limits on pasteurized and raw milk

After comparing the recovery rates of four different viruses (IAV H3N2, inactivated HPAI H5N1, PEDV, and MgV) and optimizing the nucleic acid extraction method, the limits of detection at 50 and 95 % confidence intervals (LoD_50%_ and LoD_95%_, respectively) were determined by detecting IAV H3N2 and inactivated HPAI H5N1. These viruses were serially 10-fold diluted from 2.5 x 10^6^ to 25 genome copies (gc) and seeded into 200 mL of pasteurized and raw milk samples that had been previously tested negative. For the determination of detection limits, inoculated samples were concentrated using the AlCl_3_ method and subjected to nucleic acid extraction with the Maxwell^®^ RSC Instrument (Promega) using the Maxwell RSC Pure Food GMO and authentication kit (Promega), including a pretreatment with Plant RNA Isolation Aid, as described in the section above. All experiments were performed in triplicate, with three independent milk samples concentrated for each inoculation level, following the statistical approach described by Wilrich and Wilrich (2009). Viral detection was performed by the RT-qPCR Assay A.

### 2.5 Assessment of intercalating markers for capsid integrity RT-qPCR

Propidium monoazide (PMAxx™) (Biotium, Freemont, USA), platinum (IV) chloride (PtCl_4_) (Acros Organics) and the Viability PCR Crosslinker (CL) (Promega) were evaluated as intercalating markers. Capsid integrity pre-treatments were performed in DNA LoBind 1.5 mL tubes. Samples were treated with PMAxx™ (100 μM) and 0.5 % Triton X-100 (ThermoFisher Scientific) and incubated in the dark for 30 min on an orbital shaker (150 rpm) at RT. To photoactivate PMAxx™ and facilitate its covalent binding to viral RNA, the sample was exposed to blue LED light for 15 min using the PMA-Lite™ LED Photolysis Device (Biotium) (Kevill et al., 2024), followed by 15 min of incubation in darkness, and an additional 15 min photoactivation cycle (Puchades-Colera et al., 2024; Randazzo et al., 2018). Alternatively, capsid integrity treatments with PtCl_4_ were performed by incubating samples for 30 min with a 2.5 mM PtCl_4_ at RT (Cuevas-Ferrando et al., 2021). Additionally, samples were incubated for 30 min at 37 °C with 50 μM CL, followed by a vortexing step and 30 min incubation at 37 °C. Finally, 20 μL of neutralization buffer (Promega) was added, and samples were incubated for 15 min at RT (Wales et al., 2024).

The three intercalating markers [PMAxx™ (100 μM), CL (50 μM) and PtCl_4_ (2.5 mM)] were initially tested on the Twist Synthetic InfluenzaV H1N1 RNA control (Twist Bioscience). Each experiment included a synthetic RNA suspension without intercalating markers as a positive control and was detected by the RT-qPCR Assay A.

To further evaluate the performance of the intercalating markers, IAV H3N2 suspensions in PBS were heat-treated at 99 °C for 5 min. Then, 300 μL of both heat-treated and non-inactivated IAV H3N2 suspensions were incubated in *DNA* LoBind tubes with each of the intercalating agents (100 μM PMAxx™, 2.5 mM PtCl_4_ or 50 μM CL). Following the capsid integrity pre-treatments, viral genomes were immediately extracted using the previously described method with the addition of Plant RNA Isolation Aid and subsequently detected by RT-qPCR. To minimize the interference of the milk matrix (i.e., fat, lactose, proteins, calcium ions) with the intercalation reaction and subsequent photoactivation (particularly for PMAxx™, a photoactivable dye), the effect of sample dilution was investigated. Concentrated milk samples were tested in non-diluted, 2-fold and 5-fold diluted forms.

After optimizing the capsid integrity pretreatment conditions for IAV H3N2, the three intercalating markers (PMAxx™ (100 μM), CL (50 μM) and PtCl_4_ (2.5 mM)) were further evaluated using purified HPAI H5N1 RNA in PBS. These treated samples were then analyzed using the three RT-qPCR assays (assays A, B, and C; section 2.2) to comprehensively evaluate the performance of each marker. Finally, capsid integrity RT-qPCR was performed using two heat-inactivated HPAI H5N1 viral suspensions (70 °C for 1 h and 99 °C for 5 min), inoculated in 5-fold diluted pasteurized and raw milk concentrates, to evaluate the effectiveness of the three intercalating markers in reducing amplification signal.

### 2.6 Method comparison for the detection of IAV on surfaces

Two methods were compared by artificially inoculating clean, disinfected stainless steel and food-grade silicone surfaces (10 x 10 cm) with 600 μL of virus suspensions containing heat-inactivated HPAI H5N1 (approx. 3 x 10^3^ gc) and PEDV (approx. 2.4 x 10^5^ gc). Later, recoveries using Method B were also assessed for IAV H3N2 (approx. 6 x 10^5^ gc), and MgV (approx. 6 x 10^3^ gc). Virus suspensions were prepared in either PBS or raw milk. After inoculation, surfaces were left to dry at RT inside a biosafety cabinet until the inoculum was visually dry.

The first swabbing method (Method A) used a commercially available cellulose sampling sponge (Sponge-Stick^®^, 3M, St. Paul, USA). The sponge was pre-hydrated with 2 mL of PBS and used to wipe the contaminated surface following a systematic pattern: vertically, then horizontally after turning the sponge, and finally diagonally using the sides, as described by Rose et al., 2011. After swabbing, the sponge heads were placed into sealable bags, and the handles were aseptically removed. Subsequently, 4 mL of PBS was added to each bag, and the sponges were manually compressed to release the absorbed liquid.

The second swabbing method (Method B) followed the recommendations of the ISO 15216-1:2017 standard. In brief, contaminated surfaces were swabbed intensively using a sterile, cotton-headed, plastic-shaft swab moistened with PBS, applying a moderate pressure to detach the viral particles. The swab was then immediately immersed in a tube containing 500 μL of PBS, vortexed, and pressed against the side of the tube to release the liquid. A 300 μL of the resulting eluate for each of the methods was collected for nucleic acid extraction, following the protocol described above.

### 2.7 Statistical analysis

All statistical analyses were performed using RStudio version (2023.04.21) running R version 4.3.0, with statistical significance set at *p*-value < 0.05. Data was obtained from three independent experiments with at least two technical replicates for each variable. The data was tested for normality distribution using the Shapiro-Wilk test with a 95% confidence interval. The assumption of homogeneity of variances was assessed using Levene's test to verify the equality of variances across groups. The analytical performance of the two extraction protocols in the three milk matrices was evaluated by comparing mean virus recoveries. For IAV H3N2, IAV H5N1 and MgV, comparisons were performed using the non-parametric Mann–Whitney *U* test, followed by Dunn's multiple comparison test with Bonferroni correction. For PEDV, a two-way ANOVA was conducted to assess the effects of extraction protocol and milk matrix, followed by Tukey's Honest Significant Difference (HSD) test for *post hoc* multiple comparisons.

Once the extraction protocol was selected, the average recovery of the three viruses was compared across the different milk matrices. For IAV H3N2 and PEDV, the Kruskal–Wallis test was applied, followed by Dunn's test with Bonferroni correction. In the case of MgV, a one-way ANOVA was performed, followed by Tukey's HSD test. Also, to assess whether the coefficient of variation (CV) for raw milk is significantly higher than for pasteurized and UHT milk, *post-hoc* comparisons within each extraction method were performed using Tukey's HSD test following a two-way ANOVA.

Differences in performance of the intercalating markers was evaluated by comparing mean reductions, while differences between swabbing methods and the influence of surface type and matrices (stainless steel vs. food-grade silicone) was analyzed by the comparison of viral mean recoveries. Both analyses were assessed using one-way ANOVA with Tukey's HSD test.

## 3 Results and discussion

### 3.1 Comparative analysis of methods for milk sample testing

Several studies have evaluated procedures for extracting viral nucleic acids from milk samples, including methods targeting IAV (Sayed et al., 2020; Snoeck et al., 2024; Stachler et al., 2025; Suarez et al., 2025; Zulli et al., 2024). However, to our knowledge, no studies have specifically investigated methods for concentrating IAV in milk samples, a potentially critical step when viral loads are expected to be low, as in bulk tank milk. Therefore, our initial objective was to evaluate the performance of a concentration method suitable for application in milk samples.

**Figure 1:**
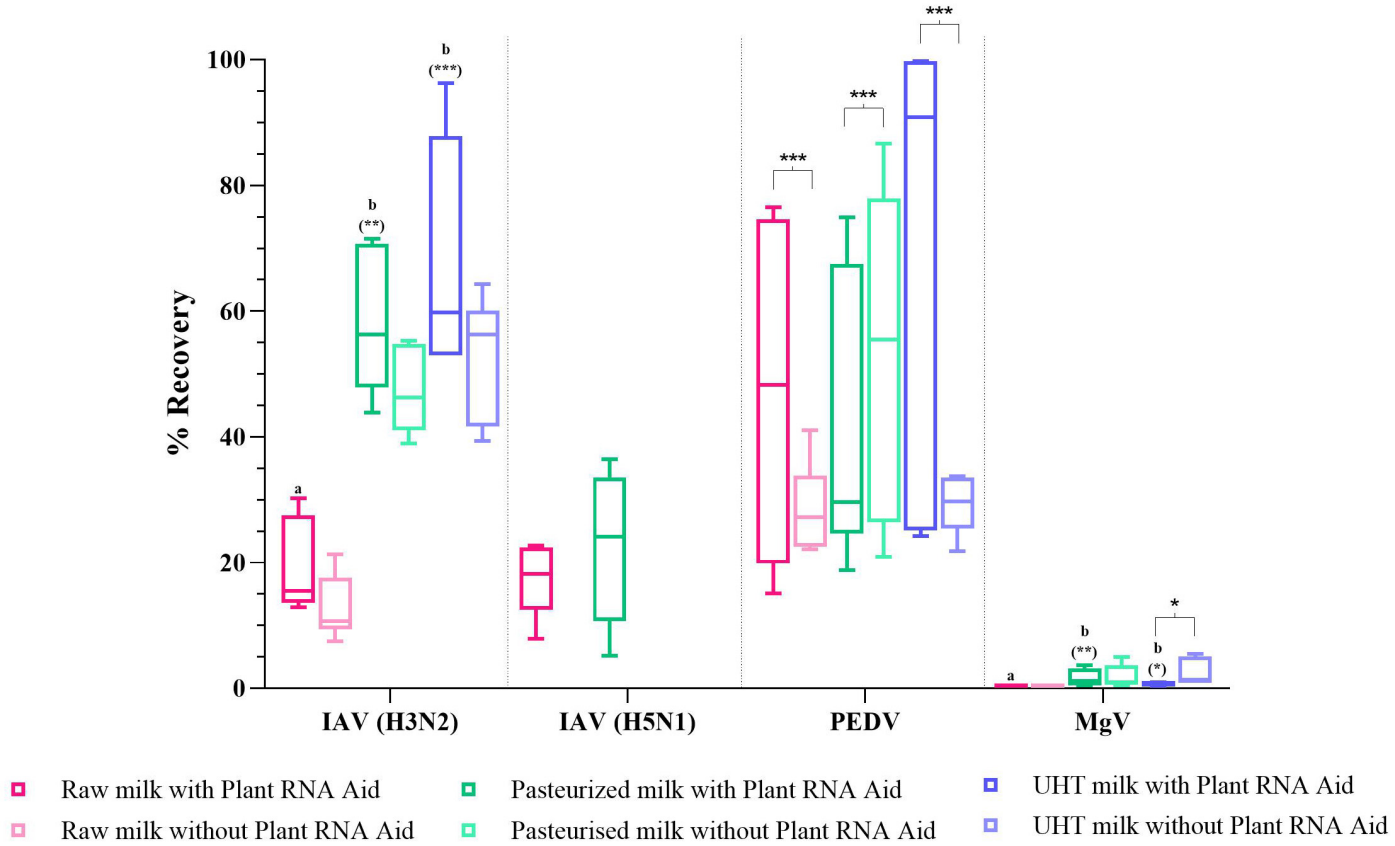
Recovery rates of the three viruses analyzed in ultra-high temperature (UHT), pasteurized, and raw milk with (dark colors) and without (soft colors) the addition of Plant RNA Isolation Aid (Thermo Fisher) in the extraction protocol. Different letters indicate significant differences in mean recovery rates of the same virus across milk types (*p* < 0.05). Significance levels: *, 0.01 ≤ *p* < 0.05; **, 0.001 ≤ *p* < 0.01; ***, *p* < 0.001 IAV, influenza A virus; PEDV, porcine epidemic diarrhea virus; MgV, mengovirus.

Milk samples were initially processed using the AlCl_3_ method (AAVV, 2018; Girón-Guzmán et al., 2024; Randazzo et al., 2020) to evaluate its effectiveness in concentrating IAV H3N2 from UHT, pasteurized, and raw milk. Nucleic acids were extracted using the Maxwell^®^ RSC Instrument with the Pure Food GMO and Authentication Kit, and the use of Plant RNA Isolation Aid was also evaluated. Recovery efficiency for each milk type was assessed by using a generic RT-qPCR for IAV (Assay A). Mean recoveries of IAV H3N2 across the three types of milk ranged from 19.09 ± 7.41 % to 67.78 ± 18.29 % with Plant RNA Isolation Aid, and from 12.75 ± 5.11 % to 52.87 ± 9.76 % without it (Figure 1). A 10-fold dilution of each RNA sample was also analyzed, showing no significant inhibition when using the Plant RNA Isolation Aid (data not shown). Using both approaches, with or without Plant RNA Isolation Aid, the mean recoveries in raw milk were significantly lower (*p* < 0.05) compared to those in pasteurized and UHT milk. Specifically, recoveries were 12.75 ± 5.11 % without the aid and 19.09 ± 7.41 % with it. In addition, raw milk samples exhibited higher coefficients of variation (CVs of 38.80 and 40.11 %, with and without aid, respectively), indicating greater variability than observed in pasteurized (19.43 and 14.57 %, with and without aid, respectively) and UHT milk (26.99 and 18.46 %, with and without aid, respectively). There were no significant differences in the mean recoveries of IAV H3N2 between pasteurized and UHT milk. These results emphasize that the composition of the dairy matrix, especially its fat content and constituents like lactose, caseins, whey proteins, and calcium ions, can significantly affect virus concentration and isolation (Bickley et al., 1996; Yavarmanesh et al., 2013, 2015).

PEDV, an enveloped virus model, showed the highest mean recovery rates across all milk types, ranging from 28.63 ± 6.97 to 72.53 ± 37.30 % (Figure 1). This method showed limited effectiveness for MgV, a non-enveloped virus model, with recovery rates ranging from 0.18 ± 0.08 to 2.45 ± 2.13 % without the aid and from 0.25 ± 0.15 to 1.66 ± 1.37 % with it. This contrasts with previous studies, where the AlCl_3_ precipitation method demonstrated successful recovery of MgV and human enteric viruses from sewage samples (Girón-Guzmán et al., 2023, 2024), suggesting the need for further optimization in milk matrices.

According to ISO 15216-1:2017 criteria, which require a minimum virus recovery rate > 1 % for reliable analysis in food samples, the AlCl_3_-based concentration method is considered satisfactory for the concentration of IAV from cow's milk. Although the addition of Plant RNA Isolation Aid did not significantly improve recovery for H3N2, it did help reduce inhibitors in raw milk samples. The improved recovery observed with the other two viral models justified its inclusion in subsequent experiments to standardize the method across different virus types.

To evaluate the effectiveness of the concentration method for the HPAI strain H5N1, currently of concern to the dairy industry, mean recoveries of heat-inactivated HPAI H5N1 were tested in both pasteurized and raw milk samples using Plant RNA Isolation Aid. In raw milk, HPAI H5N1 recoveries were similar to those observed for the IAV H3N2 strain (Figure 1), whereas in pasteurized milk, HPAI H5N1 recoveries were significantly lower (*p* < 0.05). Specifically, the mean recovery rates for HPAI H5N1 were 22.46 ± 11.86 % in pasteurized milk and 17.22 ± 6.15 % in raw milk (Figure 1). However, the CV was higher in pasteurized milk (52.82 %) compared to that for IAV H3N2 (19.43 %), while the CVs obtained for both viruses in raw milk were similar.

### 3.2 Limits of detection of IAV H3N2 and H5N1 in pasteurized and raw milk

The detection limits of IAV subtypes H3N2 and H5N1 in both pasteurized and raw milk were evaluated by targeting the M gene (Assay A), using milk samples that were serially spiked with virus suspensions. The limits of detection (LoD_95%_ and LoD_50%_) were determined by processing 200 mL of inoculated milk using the AlCl_3_-based concentration method, followed by nucleic acid extraction with the Maxwell platform and Plant RNA Isolation Aid. In pasteurized milk, the LoD_95%_ values were 8.13 × 10^4^ gc/L for IAV H3N2 and 3.10 × 10^5^ gc/L for HPAI H5N1. In raw milk, the corresponding LoD_95%_ values were 2.90 × 10^5^ gc/L for IAV H3N2 and 1.20 × 10^4^ gc/L for HPAI H5N1 (Table 1).

**Table 1:** Limit of detection (LoD_95%_ and LoD_50%_) of IAV H3N2 and H5N1 in pasteurized and raw milk processed with the selected method and detected by using the generic RT-qPCR for influenza A virus (assay A).

		Levels of inoculated virus (gc/200 mL)							
Virus	Type of milk	2.5 x 10^6^	2.5 x 10^5^	2.5 x 10^4^	2.5 x 10^3^	2.5 x 10^2^	2.5 x 101	LoD_95%_ (gc/L)	LoD_50%_ (gc/L)
H3N2	Pasteurized	6/6	6/6	6/6	2/6	0/6	0/6	8.13x10^4^	1.90x10^4^
	Raw	6/6	6/6	3/6	3/6	0/6	0/6	2.90x10^5^	6.70x10^4^
H5N1	Pasteurized	6/6	6/6	4/6	1/6	0/6	0/6	3.10x10^5^	7.17x10^4^
	Raw	6/6	6/6	6/6	6/6	1/6	0/6	1.20x10^4^	2.79x10^3^

gc, genome copies.

Several extraction methods for detecting HPAI H5N1 have been evaluated, however, the LoD_95%_ has only been established by Stachler et al. (2025) at 10^7^ gc/L and Hsiao et al. (2025), reaching a detection limit of up to 10^5^ gc/L. Regarding LoD_95%_ after a concentration step, no data are currently available for IAV. Nevertheless, our results demonstrate improved sensitivity compared to those reported for human viruses in milk, where LoD_95%_ were 2.8 × 10^6^ gc/L for HEV, 1.6 × 10^6^ gc/L for HAV, 8.0 × 10^5^ gc/L for norovirus GI, 1.1 × 10^6^ gc/L, for norovirus GII, and 2.8 × 10^7^ gc/L for tick-borne encephalitis virus (Hennechart-Collette et al., 2022, 2023). Altogether, these findings demonstrate that the described procedure improves the detection limits for HPAI H5N1 in milk by enabling the processing of larger sample volumes. This improvement is particularly relevant for monitoring the virus in bulk milk tanks. Moreover, our approach theoretically allows detection of viral concentrations within, or even below, the range of infectious HPAI H5N1 levels shed by infected cattle (10^4^-10^8.8^ TCID_50_/mL) (Caserta et al., 2024; Le Sage et al., 2024; Nelli et al., 2024). Considering that viral genomes may be partially degraded in commercially available dairy matrices after thermal processing, and that the levels as low as 10^3^ TCID_50_/mL have been reported in retail sample (Spackman et al., 2024a), analyzing larger sample volumes could further enhance the method's sensitivity.

### 3.3 Performance of capsid integrity RT-qPCR on IAV H3N2 and HPAI H5N1

Since December 2024, the USDA has initiated its national pooled milk testing for raw milk, following previous studies that detected H5N1 in both raw and retail pasteurized milk samples (USDA, 2024). This strategy has been implemented as a surveillance program to identify affected herds and respond accordingly. However, some food safety concerns have been raised following the detection of H5N1 in retail pasteurized milk and dairy products (Lail et al., 2025; Lauring et al., 2024; Spackman et al., 2024a; Suarez et al., 2025; Wallace et al., 2025a,b), although there is currently no evidence that these products contain infectious virus (Spackman et al., 2024a; Tarbuck et al., 2026).

**Figure 2:**
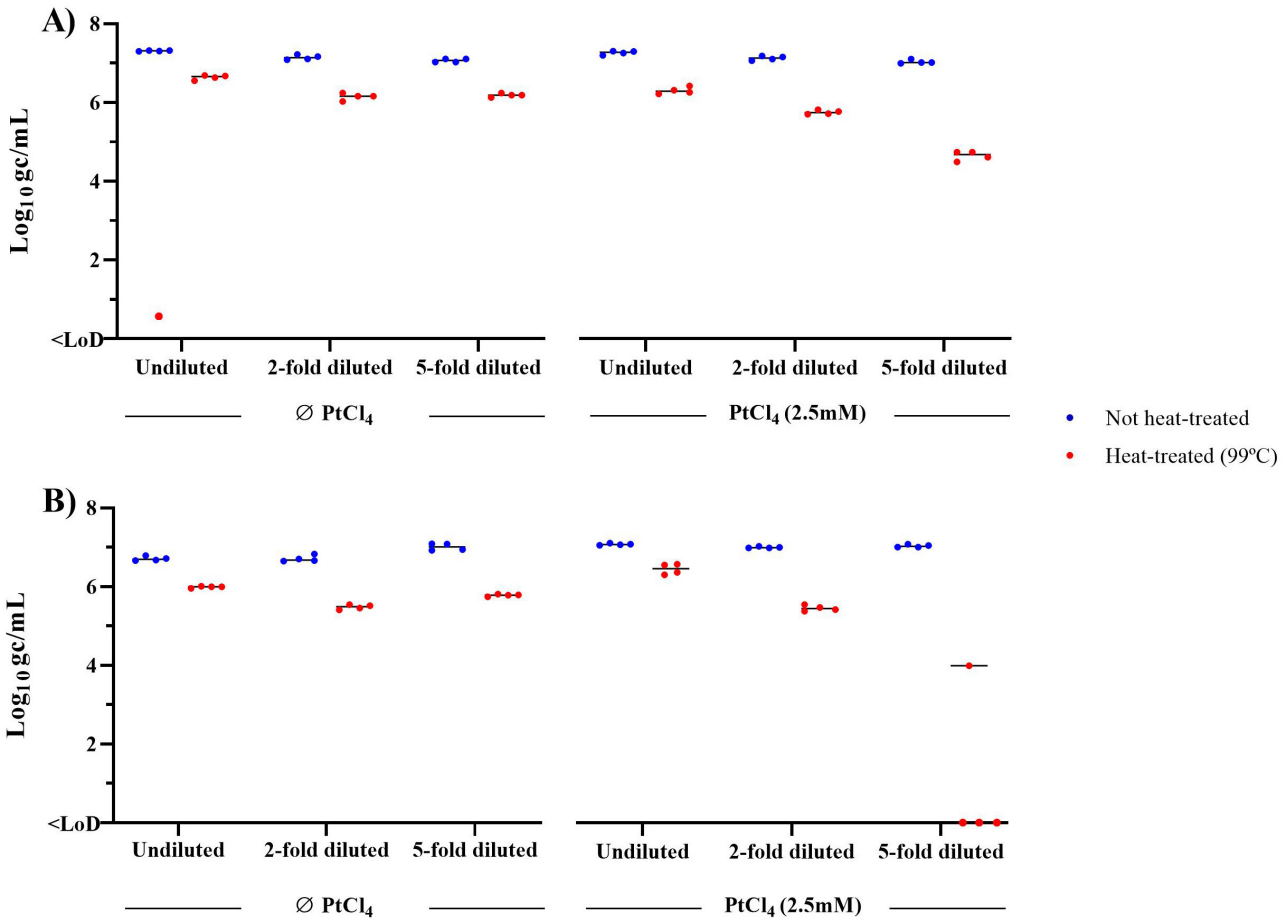
Performance of PtCl_4_ (2.5 mM) to discriminate control (not heat-treated) and heat-treated (99 °C for 5 min) IAV H3N2 suspensions artificially inoculated into pasteurized [panel **(A)**] and raw milk [panel **(B)**] concentrates (non-diluted, two-fold diluted, and 5-fold diluted) using RT-qPCR Assay A. gc, genome copies; <LoD, below the limit of detection.

RT-qPCR is the primary method used to detect IAV in milk, but it does not necessarily reflect viral infectivity, which is traditionally assessed through cell culture. Although molecular methods are useful, their limitations hinder routine application in food testing. Combining RT-qPCR with intercalating markers has recently emerged as a promising approach to rapidly assess potential infectivity of IAV and related viruses in milk and dairy products. As an initial approach to evaluate the analytical performance of intercalating markers for capsid integrity RT-qPCR in milk samples, capsid integrity pre-treatments with PMAxx™ (100 μM combined with 0.5 % Triton 100-X), PtCl_4_ (2.5 mM), and CL (50 μM) were compared using control and heat-treated (99 °C for 5 min) IAV H3N2 suspensions in PBS. Control IAV H3N2 suspensions were included in all assays, and no reduction in signal was observed, indicating that the intercalating marker did not directly affect viral particle infectivity. Furthermore, signal reduction between control and heat-treated suspensions was mainly caused by heat treatment effect on RNA integrity (Figure 2). Due to the low performance of the PMAxx™ pre-treatment (data not shown), the protocol was optimized by eliminating Triton 100-X and one of the photoactivation steps. Preliminary results indicated that PtCl_4_ (2.5 mM) significantly outperformed PMAxx™ (100 μM) and CL (50 μM) (*p* < 0.05) in distinguishing heat-treated IAV H3N2 from the control suspension, with observed Log_10_ gc/mL reductions of 3.47 ± 0.63 in 2 out of 6 replicates for PtCl_4_ (2.5 mM). Our PMAxx™ results diverge from those reported by Liang et al. (2024), a discrepancy that may be ascribed to the use of HA-specific primers or different assay conditions (i.e., PMAxx™ concentration, incubation at 37 °C rather than RT, different light sources), as described by Leifels et al. (2021). Nonetheless, and in agreement with our observations, previous investigations have demonstrated that platinum-based compounds (PtCl_4_) possess a greater capacity to bind viral genomes than monoazide reagents such as PMAxx™ (Canh et al., 2022; Cuevas-Ferrando et al., 2021). Subsequently, the performance of the capsid integrity pretreatment in pasteurized and raw milk concentrates was evaluated exclusively using PtCl_4_ (2.5 mM) due to the low performance of PMAxx™ (100 μM) and CL (50 μM). However, since PtCl_4_ exhibited lower performance in milk concentrates compared to PBS samples, dilution of the milk concentrates was applied as a previously established strategy to improve capsid integrity pretreatments in other matrices (Cuevas-Ferrando et al., 2021; Puchades-Colera et al., 2024; Randazzo et al., 2018).

Overall, in pasteurized milk concentrates, PtCl_4_ showed no reduction in undiluted samples, a reduction of 0.43 ± 0.05 Log_10_ gc/mL in all 2-fold diluted samples (4/4) and a reduction of 1.57 ± 0.17 Log_10_ gc/mL (4/4) 5-fold diluted samples. A similar trend was observed in raw milk concentrates, with the highest reduction (3.41 ± 0.64 Log_10_ gc/mL) recorded at 5-fold dilution (1/4) (Figure 2). These results provide evidence of matrix-associated inhibition (Burrough et al., 2024; Giménez-Lirola et al., 2024; Snoeck et al., 2024), highlighting the impact of milk components on the efficacy of the capsid integrity RT-qPCR treatment. The improved performance upon dilution suggests that reducing matrix complexity enhances treatment effectiveness.

**Figure 3:**
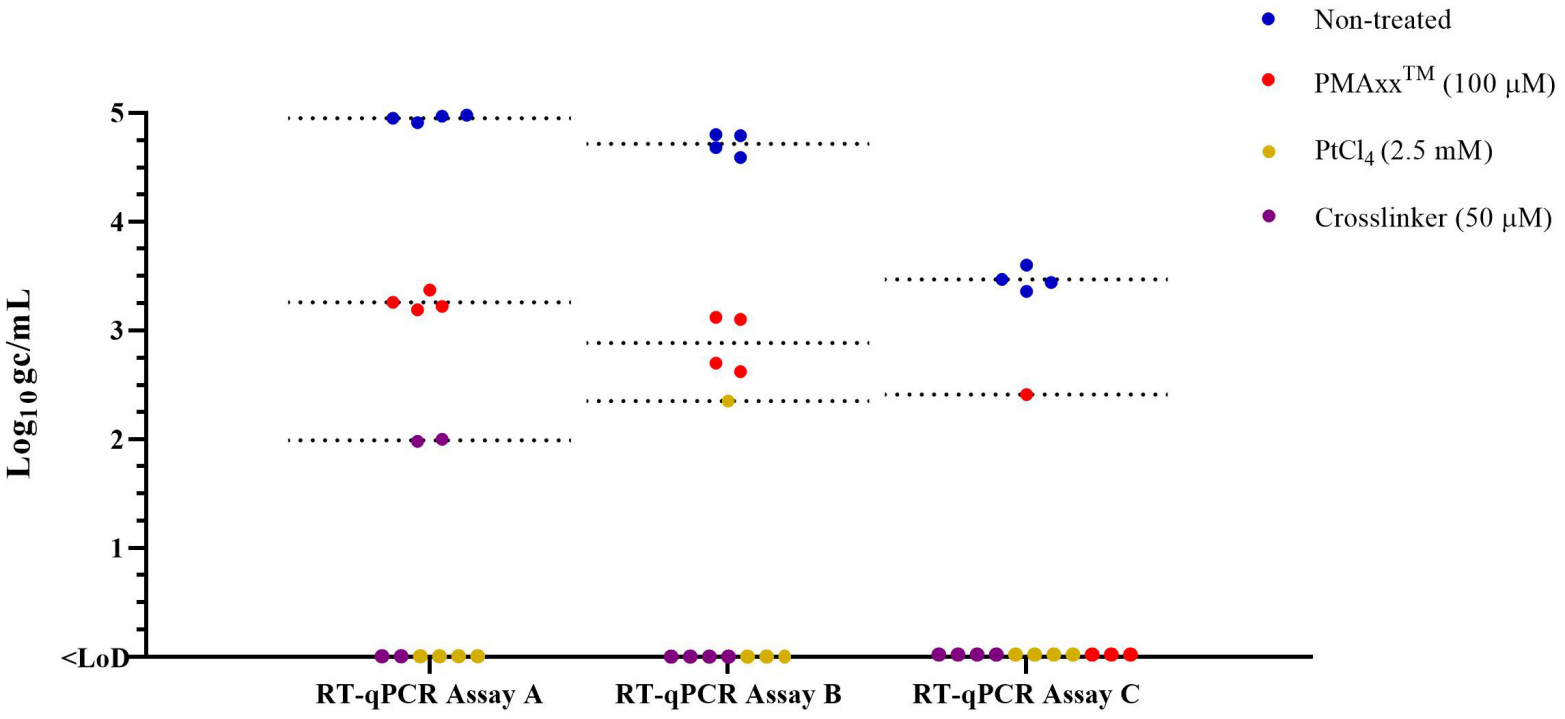
Performance of intercalating markers (PMAxx™ (100 μM), PtCl_4_ (2.5 mM) and CL (50 μM)) on HPAI H5N1 genomic RNA, assessed using three different RT-qPCR assays (Assays A, B, and C). gc, genome copies; < LoD, below the limit of detection.

The efficacy of PMAxx™ (100 μM), PtCl_4_ (2.5 mM) and CL (50 μM) in reducing the amplification signal of HPAI H5N1 purified RNA was also evaluated in PBS (Figure 3). PtCl_4_ (2.5 mM) completely prevented RNA amplification across all three RT-qPCR assays, except for one replicate using Assay B. CL (50 μM) was also highly effective, completely removing the signal except for two replicates using Assay A, where residual detection was observed. Among the three intercalating markers, PMAxx™ was the least effective in removing or reducing the RT-qPCR signal (Figure 3). Additionally, the performance of the three RT-qPCR assays (A, B, and C) was assessed, as capsid integrity RT-qPCR outcomes may vary based on factors such as the target fragment and amplicon size (Canh et al., 2022; Leifels et al., 2021). Due to the lower performance of Assay C (Figure 3) and its high limit of detection (Supplementary Table S1), assays A and B were selected for subsequent experiments involving HPAI H5N1.

**Figure 4:**
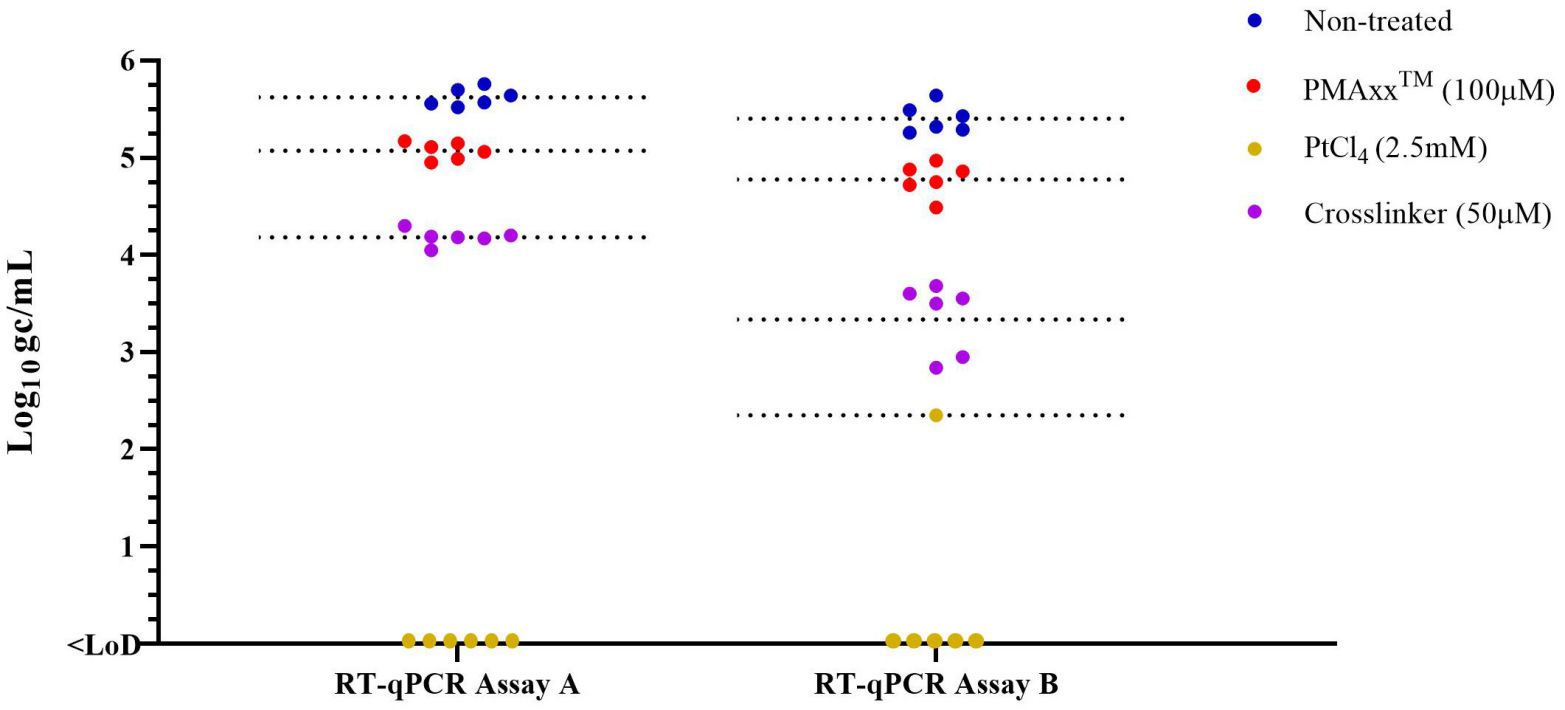
Assessment of intercalating markers (PMAxx™ (100 μM), PtCl_4_ (2.5 mM), and CL (50 μM)) on heat-inactivated HPAI H5N1 (70 °C for 1 h) suspended in PBS (*n* = 3), with RT-qPCRs assays A and B. gc, genome copies; <LoD, below the limit of detection.

We further assessed the effects of the three intercalating markers on heat-inactivated HPAI H5N1 in PBS. PMAxx™ pre-treatment (100 μM, without Triton 100-X) of heat-inactivated HPAI H5N1 suspensions (70 °C for 1 h) minimally reduced the PCR signal by 0.55 ± 0.09 and 0.62 ± 0.20 Log_10_ gc/mL (Figure 4) across both RT-qPCR assays (A and B), respectively. In contrast, CL (50 μM) demonstrated greater signal reduction, similar to results observed with purified HPAI H5N1 RNA. Specifically, CL treatment resulted in a reduction of 2.05 ± 0.43 Log_10_ gc/mL with RT-qPCR Assay B, compared to 1.44 ± 0.14 Log_10_ gc/mL reported with the RT-qPCR Assay A (Figure 4). While all intercalating markers showed statistically significant differences (*p* < 0.05) in titer reduction compared to the heat-inactivated control, only PtCl_4_ (2.5 mM) consistently achieved complete PCR signal suppression for both RT-qPCR assays.

**Figure 5:**
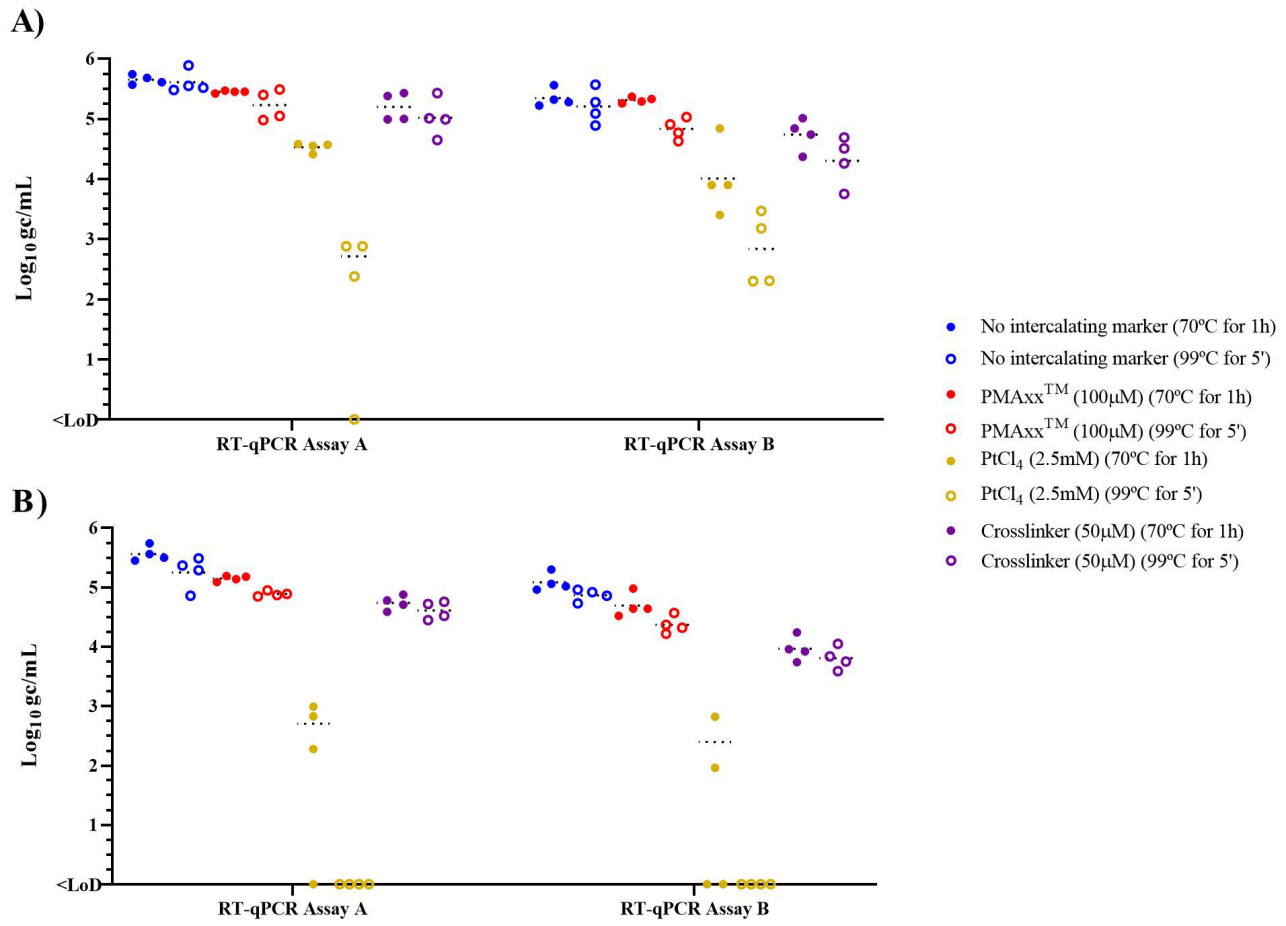
Evaluation of intercalating markers (PMAxx™ (100 μM), PtCl_4_ (2.5 mM), and CL (50 μM)) on heat-inactivated HPAI H5N1 (70 °C for 1 h and 95 °C for 5 min) suspended on 5-fold diluted pasteurized [panel **(A)**] and raw milk concentrates [panel **(B)**], with RT-qPCRs assays A and B. gc, genome copies; <LoD, below limit of detection.

Several studies have reported varying prevalence rates of HPAI H5N1 in retail pasteurized milk products using molecular methods (Giménez-Lirola et al., 2024; Lail et al., 2025; Lauring et al., 2024; Spackman et al., 2024a,b; Stachler et al., 2025; Suarez et al., 2025; Tarbuck et al., 2026; Wallace et al., 2025a). Additionally, it has been demonstrated that while HPAI H5N1 genome copy numbers measured by RT-qPCR decrease by < 1 Log following pasteurization, infectivity is effectively eliminated (Guan et al., 2024; Kaiser et al., 2024). Therefore, developing a rapid tool to assess potential infectivity in milk would greatly benefit the dairy industry and provide critical information for health authorities. To explore this, the applicability of this approach to HPAI H5N1 in milk samples was assessed by 5-fold diluting milk concentrates, based on findings from IAV H3N2. When HPAI H5N1 was inactivated at 70 °C for 1 hour, only PtCl_4_ (2.5 mM) significantly reduced (*p* < 0.05) the RT-qPCR signal, with decreases of 1.12 ± 0.04 and 1.35 ± 0.27 Log_10_ gc/mL in pasteurized milk, and reductions of 2.97 ± 0.39 and 3.70 ± 1.55 Log_10_ gc/mL in raw milk, as measured by RT-qPCR assays A and B, respectively (Figure 5).

Given that several studies (Canh et al., 2022; Leifels et al., 2021) have shown that the efficacy of intercalating markers can vary depending on the inactivation treatment, a more intense heat treatment (99 °C for 5 min) was applied to HPAI H5N1 suspensions to reassess the performance of the intercalating markers. Overall, the results showed that under extreme heat treatment conditions, pretreatment with PtCl_4_ (2.5 mM) was highly effective in raw milk, completely eliminating the signal in both RT-qPCR assays. In pasteurized milk samples, an average reduction of 2.84 ± 0.01 and 2.19 ± 0.37 Log_10_ gc/mL was observed for RT-qPCR assays A and B, respectively (Figure 5). Although intercalating markers have shown promise for detecting some human enteric viruses and SARS-CoV-2 (Cuevas-Ferrando et al., 2021; Girón-Guzmán et al., 2025; Puchades-Colera et al., 2024; Randazzo et al., 2018) in various matrices, our results indicate that this pretreatment is only useful for assessing HPAI H5N1 infectivity in milk samples under extreme processing conditions, limiting its broader applicability. Nevertheless, it effectively removes free RNA from samples, making it a potential tool for reducing the number of positive samples that require testing in embryonated chicken eggs or MDCK cells (Guan et al., 2024).

### 3.4 Evaluation of methods for virus detection on surfaces

Surface testing for HPAI H5N1 on dairy farms is crucial for early detection and containment, helping to prevent viral spread, ensure milk safety, and protect both animal and human health within the dairy industry. Therefore, this study initially focused on evaluating two swabbing methods, one based on a commercially available sponge (Method A) and the other following the ISO 15216:1 procedure (Method B). The viral recovery efficiency of Method A was tested for both HPAI H5N1 and PEDV. Results showed low recovery rates when viruses were diluted in PBS, ranging from 0.10 to 0.60 % for H5N1 and from 2.57 to 9.42% for PEDV. When viral suspensions were diluted in raw milk, no recovery was obtained for H5N1 while PEDV recovery was below 1%. Thus, all subsequent surface testing was performed exclusively using Method B, in accordance with the ISO 15216:1 standard. To this end, the recovery of IAV H3N2, HPAI H5N1, PEDV and MgV was evaluated on two surfaces (stainless steel and food-grade silicone).

**Figure 6:**
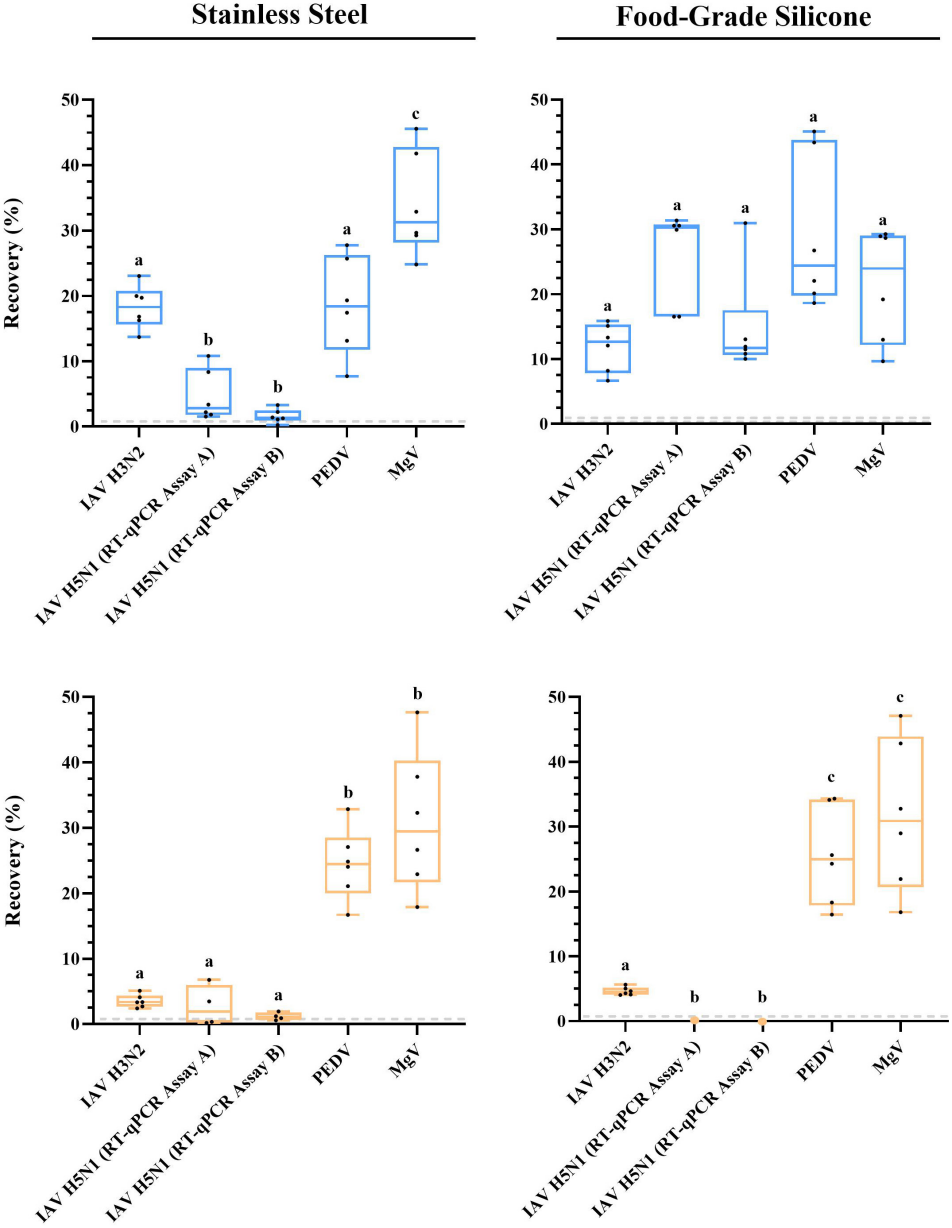
Recovery yields of IAV H3N2, HPAI H5N1 (using RT-qPCR assays A and B), PEDV and MgV from artificially contaminated stainless steel (*n* = 3) and food-grade silicone (*n* = 3) surfaces (10 cm^2^), using PBS (blue boxes) and raw milk (orange boxes) as matrix. Surface swabbing method B was based on ISO 15216:1 standard norm. Different letters show statistically differences in mean virus recovery on each type of surface. The dashed gray line represents the 1% recovery threshold recommended by ISO 15216-1 for reliable detection. PEDV, porcine epidemic diarrhea virus; MgV, mengovirus; IAV, influenza A virus.

On stainless steel, RT-qPCR Assay B did not meet the ISO 15216:1 minimum recovery threshold of 1 % for HPAI H5N1 in any technical replicate, regardless of whether the virus was prepared in PBS or raw milk (Figure 6). In contrast, RT-qPCR Assay A achieved mean recoveries of 5.13 ± 2.34% in raw milk and 4.69 ± 3.91 % in PBS.

For IAV H3N2 prepared in PBS, recovery reached 18.27 ± 3.32 %, whereas recovery was lower in raw milk, at 3.51 ± 0.97 %. Moreover, when comparing the enveloped virus model (PEDV) to the non-enveloped model (MgV), higher recoveries were consistently observed for MgV in both PBS (34.01 ± 8.03 %) and raw milk (30.87 ± 10.78 %), compared to 18.52 ± 7.55 % in PBS and 24.44 ± 5.46 % in raw milk for PEDV. Notably, MgV also exhibited a higher CV in raw milk, at 34.93 %.

On food-grade silicone surfaces, mean recoveries of HPAI H5N1 using method B were higher than those observed on stainless steel, reaching 26.86 ± 8.82 % and 14.72 ± 8.03 % in PBS viral suspensions with RT-qPCR assays A and B, respectively. However, HPAI H5N1 prepared in raw milk could not be recovered from food-grade silicone surfaces (Figure 6). In contrast to stainless steel, IAV H3N2 recoveries on silicone were lower, with mean values of 11.87 ± 3.72 % in PBS and 5.07 ± 0.66 % in raw milk suspensions. PEDV showed relatively high mean recoveries, with 29.35 ± 11.87 % in PBS and 25.52 ± 7.58 % in raw milk, along with a lower CV in raw milk. Similarly, mean recoveries of MgV were 21.46 ± 8.78 % in PBS and 31.73 ± 11.71 % in raw milk.

Surface type significantly influenced the recovery of H3N2 and H5N1 in PBS, with higher recovery of H5N1 from food-grade silicone and of H3N2 from stainless steel. However, when viruses were diluted in raw milk, no significant differences were observed for H3N2 recovery (*p* > 0.05), while H5N1 was not recovered from food-grade silicone (Figure 6). The matrix effect was evaluated by comparing viral recoveries from PBS and raw milk on different surfaces (Figure 6 and Supplementary Table S2). Notably, food-grade silicone surfaces showed a significant decrease in viral recovery when raw milk was used. In these cases, H5N1 was undetectable, and H3N2 recovery was considerably lower (5.07 ± 0.66 %) compared to PBS (11.87 ± 3.72 %). In contrast, stainless steel surfaces did not show significant differences in H5N1 recovery between the two matrices. However, H3N2 recovery was significantly reduced in raw milk (3.51 ± 0.97 %) compared to PBS (18.27 ± 3.32 %). These findings align with previous studies showing that surface material, desiccation, and matrix composition can significantly influence viral recovery (Cox, 1993; Cuevas-Ferrando et al., 2022; Jones et al., 2020; Keeratipibul et al., 2017; Kwon et al., 2024a,b).

Taken together, our findings underscore the need for further surface recovery studies to optimize swabbing techniques and enhance viral yield, thereby contributing to a better understanding, prevention, and control of HPAI H5N1 transmission and other viral pathogens.

## 4 Conclusions

This study provides a validated method for detecting influenza A virus, including HPAI H5N1, in milk and on dairy farm surfaces. An aluminum-based concentration protocol paired with magnetic bead-based RNA extraction effectively recovered viral RNA from milk, meeting ISO 15216:1 standards, although recoveries were lower and more variable in raw milk. Notably, our method achieved LoD_95%_ that are superior to those currently reported in the literature, enhancing sensitivity for early detection. Capsid integrity RT-qPCR using PtCl_4_ was the most effective for distinguishing inactivated virus but only under extreme heat treatment conditions, limiting its use for routine infectivity assessment.

For environmental monitoring, the ISO 15216:1-compliant swabbing method (Method B) was superior to sponge-based sampling. These findings support the integration of optimized milk and surface testing protocols to improve surveillance, guide outbreak response, and reduce zoonotic risk in the dairy industry. A key limitation of this study is the use of milk artificially spiked with inactivated HPAI H5N1. As viral behavior in naturally infected milk may differ, future investigations employing naturally infected samples are essential to validate these findings.

## Data Availability

The original contributions presented in the study are included in the article/Supplementary material, further inquiries can be directed to the corresponding author.
