# Supplementary material for: Methods for detecting highly pathogenic avian influenza H5N1 virus in dairy processing environments
Source: Front Microbiol. 2026 Jun 4;17:1842786. doi: 10.3389/fmicb.2026.1842786 (PMC13275685; doi:10.3389/fmicb.2026.1842786)
Supplement: Supplementary file 1 [file Data_Sheet_1.docx]

Supplementary Material

**Methods for detecting avian influenza virus H5N1 in dairy processing environments**

Pablo Puchades-Colera ^1^, Inés Girón-Guzman^1^, Alba Pérez-Cataluña^1^, Gloria Sánchez^1^

^1^VISAFELab. Department of Preservation and Food Safety Technologies. Institute of Agrochemistry and Food Technology. IATA-CSIC. Av. Agustín Escardino 7. Paterna. 46980. Valencia. Spain.

*Corresponding author: [gloriasanchez@iata.csic.es](mailto:gloriasanchez@iata.csic.es), [alba.perez@iata.csic.es](mailto:alba.perez@iata.csic.es)

**Supplementary Table S1.** Primers, probes, RT-qPCR conditions, limit of quantification (gc/reaction) and limit of detection (gc/reaction) for all targeted viruses included in this work. Abbreviations: gc, genome copies; MgV, mengovirus; PEDV, porcine epidemic diarrhea virus; IAV, influenza A virus; LoQ, limit of quantification; LoD, limit of detection.

| **Virus** | **Primers+ Probe** | **Sequence (5’🡪3’)** | **PCR Conditions** | | **LoQ (gc/reaction)** | **LoD (gc/reaction)** | **Reference** |
| --- | --- | --- | --- | --- | --- | --- | --- |
| **MgV** | Forward | GCGGGTCCTGCCGAAAGT | 42°C, 15min |  | 73.65 | 51.91 | ISO 15216-1:2017 |
|  | Reverse | GAAGTAACATATAGACAGACGCACAC | 95°C, 2 min |  |  |  |  |
|  | Probe | ATCACATTACTGGCCGAAGC | 95°C, 15sec | x45 cycles |  |  |  |
|  |  |  | 60°C, 60sec |  |  |  |  |
|  |  |  |  |  |  |  |  |
| **PEDV** | Forward | CAGGACACATTCTTGGTGGTCTT | 42°C, 15min |  | 15.54 | 10.96 | Zhou et al., (2017) |
|  | Reverse | CAAGCAATGTACCACTAAGGAGTGTT | 95°C, 2min |  |  |  |  |
|  | Probe | ACGCGCTTCTCACTAC | 95°C, 15sec | x45 cycles |  |  |  |
|  |  |  | 60°C, 60sec |  |  |  |  |
|  |  |  |  |  |  |  |  |
| **IAV** | Forward1 | CAAGACCAATCYTGTCACCTCTGAC | 50°C, 30min |  | 6.22 | 3.91 | CDC (2021) |
| **(assay A)** | Forward2 | CAAGACCAATYCTGTCACCTYTGAC | 95°C, 2min |  |  |  |  |
|  | Reverse1 | CGATTYTGGACAAAVCGTCTACG | 95°C, 15sec | x45 cycles |  |  |  |
|  | Reverse2 | GCATTTTGGATAAAGCGTCTACG | 55°C, 30sec |  |  |  |  |
|  | Probe | TGCAGTCCT/ZEN/CGCTCACTGGGCACG |  |  |  |  |  |
|  |  |  |  |  |  |  |  |
| **IAV H5** | Forward | TATAGARGGAGGATGGCAGG | 50°C, 30min |  | 3.64 | 1.22 | Wolfe et al., (2024) |
| **(assay B)** | Reverse | ACDGCCTCAAAYTGAGTGTT | 95°C, 2min |  |  |  |  |
|  | Probe | AGGGGAGTGGKTACGCTGCRGAC | 95°C, 30sec | x45 cycles |  |  |  |
|  |  |  | 59°C, 30sec |  |  |  |  |
|  |  |  |  |  |  |  |  |
| **IAV H5**  **clade 2** | Forward | CGATCTTAATGGAGTGAAGCCCC | 50°C, 30min |  | 19.92 | 10.20 | WHO (2021) |
| **(assay C)** | Reverse | CCCTCTCCACGATGTAAGACCATTC | 95°C, 2min |  |  |  |  |
|  | Probe1 | AGCCAYCCAGCTACRCTACA | 95°C, 30sec | x45 cycles |  |  |  |
|  | Probe2 | AGCCATCCCGCAACACTACA | 59°C, 30sec |  |  |  |  |
|  |  |  |  |  |  |  |  |

**Supplementary Table S2.** Mean recovery percentages and coefficients of variation (CV) of IAV H3N2, HPAI H5N1, PEDV, and MgV analysed in ultra-high temperature (UHT), pasteurized, and raw milk concentrated using the AlCl_3_ method. Nucleic acids were extracted with the beads-based extraction protocol, either without or with Plant RNA Isolation Aid. Values between brackets indicates recovery ranges. Different letters indicate significant differences between recoveries achieved with both methods. Abbreviations: IAV, influenza A virus; PEDV, porcine epidemic diarrhea virus; MgV, mengovirus; HPAI, highly pathogenic avian influenza; Nd, not determined.

| Milk type | Virus | Without Plant RNA Isolation Aid | |  | With Plant RNA Isolation Aid | |
| --- | --- | --- | --- | --- | --- | --- |
|  |  | **Recovery** | **CV (%)** |  | **Recovery** | **CV (%)** |
| UHT | **IAV**  **H3N2** | 52.87 ± 9.76^a^  (39.36 – 64.22) | 18.46 |  | 67.78 ± 18.29^a^  (52.96 – 96.21) | 26.99 |
|  | **PEDV** | 29.20 ± 4.49^a^  (21.84 – 33.78) | 15.37 |  | 72.53 ± 37.30^b^  (24.22 – 104.12) | 51.43 |
|  | **MgV** | 2.45 ± 2.13 ^a^  (1.04 – 5.50) | 8.70 |  | 0.66 ± 0.22 ^b^  (0.39 – 1.00) | 32.78 |
| Pasteurised | **IAV**  **H3N2** | 47.17 ± 6.87^a^  (38.94 – 55.24) | 14.57 |  | 57.90 ± 11.25^a^  (43.87 – 71.57) | 19.43 |
|  | **HPAI**  **H5N1** | Nd | - |  | 22.46 ± 11.86  (5.12 – 36.40) | 52.82 |
|  | **PEDV** | 53.65 ± 25.68 ^a^  (20.90 – 86.68) | 47.87 |  | 40.77 ± 23.27 ^b^  (18.73 – 74.94) | 57.07 |
|  | **MgV** | 1.86 ± 1.84^a^  (0.48 – 4.97) | 9.88 |  | 1.66 ± 1.37^a^  (0.45 – 3.69) | 82.87 |
| Raw | **IAV**  **H3N2** | 12.75 ± 5.11^a^  (7.47 – 21.32) | 40.11 |  | 19.09 ± 7.41^a^  (12.87– 30.27) | 38.80 |
|  | **HPAI**  **H5N1** | Nd | - |  | 17.22 ± 6.15  (7.87 – 22.28) | 35.70 |
|  | **PEDV** | 28.63 ± 6.97 ^a^  (22.09 – 41.02) | 24.35 |  | 47.26 ± 25.76 ^b^  (15.04 – 76.52) | 54.50 |
|  | **MgV** | 0.18 ± 0.08^a^  (0.12 – 0.34) | 4.77 |  | 0.25 ± 0.15^a^  (0.09 – 0.50) | 62.77 |

**Supplementary Table S3**. Performance of PtCl_4_ (2.5mM) to discriminate control and heat-treated (99ºC for 5 min) (n=3, for each condition) IAV H3N2 suspensions artificially inoculated in PBS. The reduction in Log_10_ gc/mL was obtained by comparing non-inactivated and inactivated IAV H3N2 suspensions with and without viability treatment. Values in brackets indicate the number of positives replicates over the total number of replicates. Asterisk represents statistical differences in viral concentrations between non-inactivated and inactivated. Abbreviations: gc, genome copies.

| Treatment | Mean Log_10_ gc/mL | | Mean Log_10_ gc/mL difference | |
| --- | --- | --- | --- | --- |
|  |  |  |  |  |
|  | **Non-inactivated** | **Inactivated** | **Non-inactivated** | **Inactivated** |
| Non-treated | 7.25 ± 0.05  (6/6) | 7.07 ± 0.01  (6/6) | - | |
| PtCl_4_ | 6.35 ± 0.08  (6/6) | 4.35 ± 0.41  (2/6) | 0.90 ± 0.12 | 3.47 ± 0.63^*^ |

**Supplementary Table S4**. Performance of PtCl_4_ (2.5mM) to discriminate control and heat-treated (99ºC for 5 min) (n=2, for each condition) IAV H3N2 suspension artificially inoculated in pasteurized and raw milk concentrates (non-diluted, two-fold and five-fold diluted). The reduction in Log_10_ gc/mL was obtained by comparing non-inactivated and inactivated IAV H3N2 suspensions with and without viability treatment. Values in brackets indicate the number of positives replicates over the total number of replicates. Abbreviations: gc, genome copies; nr, no reduction.

|  | Treatment | Mean Log_10_ gc/mL | |  | Mean Log_10_ gc/mL difference | |
| --- | --- | --- | --- | --- | --- | --- |
|  |  |  |  |  |  |  |
| Sample |  | **Non-inactivated** | **Inactivated** |  | **Non-inactivated** | **Inactivated** |
| Pasteurized Milk | **Non-treated** | 7.31 ± 0.00  (4/4) | 6.64 ± 0.06  (4/4) |  | - | |
|  | **PtCl_4_** | 7.23 ± 0.02  (4/4) | 6.27 ± 0.09  (4/4) |  | 0.08 ± 0.02 | 1.04 ± 0.09 |
| Raw Milk | **Non-treated** | 6.71 ± 0.06  (4/4) | 5.99 ± 0.01  (4/4) |  | - | |
|  | **PtCl_4_** | 7.04 ± 0.02  (4/4) | 6.42 ± 0.16  (4/4) |  | nr | nr |
| Pasteurized Milk  2-fold diluted | **Non-treated** | 7.15 ± 0.07  (4/4) | 6.15 ± 0.07  (4/4) |  | - | |
|  | **PtCl_4_** | 7.09 ± 0.06  (4/4) | 5.72 ± 0.02  (4/4) |  | 0.06 ± 0.01 | 0.43 ± 0.05 |
| Raw Milk  2-fold diluted | **Non-treated** | 6.72 ± 0.05  (4/4) | 5.49 ± 0.01  (4/4) |  | - | |
|  | **PtCl_4_** | 6.96 ± 0.01  (4/4) | 5.42 ± 0.08  (4/4) |  | nr | 0.12 ± 0.02 |
| Pasteurized Milk  5-fold diluted | **Non-treated** | 7.07 ± 0.06  (4/4) | 6.19 ± 0.04  (4/4) |  | - | |
|  | **PtCl_4_** | 7.00 ± 0.04  (4/4) | 4.63 ± 0.13  (4/4) |  | 0.07 ± 0.02 | 1.57 ± 0.17 |
| Raw Milk  5-fold diluted | **Non-treated** | 7.01 ± 0.11  (4/4) | 5.78 ± 0.02  (4/4) |  | - | |
|  | **PtCl_4_** | 7.00 ± 0.01  (4/4) | 3.97  (1/4) |  | 0.01 ± 0.10 | 3.41 ± 0.64 |

**Supplementary Table S5**. Parameters of the three RT-qPCR assays and the generated standard curves by using the HPAI H5N1 genomic RNA.

| RT-qPCR Assay | A | B | C |
| --- | --- | --- | --- |
| Standard Curve | y = -3.43x + 37.40 R² = 0.9976 | y = -3.07x + 35.41 R² = 0.9889 | y = -3.34x + 39.49 R² = 0.9751 |
| Amplicon Size (bp) | 105 | 170 | 122 |
| LoD per reaction  (gc/2.5 µL) | 3.91 | 1.22 | 10.20 |
| LoQ per reaction  (gc/2.5 µL) | 6.22 | 3.64 | 19.92 |

**Supplementary Table S6**. Performance of PMAxx^TM^ (100µM), PtCl_4_ (2.5mM) and CL (50µM) intercalating markers on HPAI H5N1 genomic RNA suspended in PBS assessed by two RT-qPCRs assays (A and B). The reduction in Log10 gc/mL was obtained by comparing non-treated suspensions with viability treated HPAI H5N1 genomic RNA suspensions. Values in brackets indicate the number of positives replicates over the total number of replicates. Abbreviations: gc, genome copies; <LoD, below limit of detection.

| Treatment | Mean Log_10_ gc/mL | |  | Mean Log_10_ gc/mL reduction | |
| --- | --- | --- | --- | --- | --- |
|  | **Assay A** | **Assay B** |  | **Assay A** | **Assay B** |
| Non-treated | 4.95 ± 0.03  (4/4) | 4.72 ± 0.02  (4/4) |  | - | - |
| PtCl_4_ | <LoD  (0/4) | 2.35  (1/4) |  | 4.24 ± 0.03 | 3.55 ± 1.65 |
| PMAxx^TM^ | 3.27 ± 0.05  (4/4) | 2.94 ± 0.00  (4/4) |  | 1.69 ± 0.08 | 1.78 ± 0.02 |
| *Crosslinker* | 1.99 ± 0.02  (2/4) | <LoD  (0/4) |  | 2.96 ± 0.05 | 4.51 ± 0.02 |

**Supplementary Table S7**. Assessment of intercalating markers (PMAxx**^TM^** (100µM), PtCl_4_ (2.5mM), and CL (50µM)) on heat-inactivated HPAI H5N1 (70ºC for 1h) suspended in PBS (n=3), assessed by two RT-qPCRs assays (A and B). The reduction in Log_10_ gc/mL was obtained by comparing the results of suspensions inactivated and detected with the RT-qPCRs assays A and B, with suspensions treated with different intercalating markers. Values in brackets indicate the number of positives replicates over the total number of replicates. Abbreviations: gc, genome copies; <LoD, below limit of detection.

| Treatment | Mean Log_10_ gc/mL | |  | Mean Log_10_ gc/mL reduction | |
| --- | --- | --- | --- | --- | --- |
|  | **Assay A** | **Assay B** |  | **Assay A** | **Assay B** |
| Non-treated | 5.63 ± 0.10  (6/6) | 5.41 ± 0.13  (6/6) |  | - | - |
| PtCl_4_ | <LoD  (0/6) | <LoD  (0/6) |  | 5.39 ± 0.33 | 5.34 ± 0.07 |
| PMAxx^TM^ | 5.07 ± 0.10  (6/6) | 4.79 ± 0.15  (6/6) |  | 0.55 ± 0.09 | 0.62 ± 0.20 |
| *Crosslinker* | 4.18 ± 0.07  (6/6) | 3.36 ± 0.40  (6/6) |  | 1.44 ± 0.14 | 2.05 ± 0.43 |

**Supplementary Table S8**. Assessment of intercalating markers (PMAxx^TM^ (100µM), PtCl_4_ (2.5mM), and CL (50µM)) on heat-inactivated HPAI H5N1 (70ºC for 1h and 95ºC for 5’) suspended on 5-fold diluted pasteurized and raw milk concentrates (n=2). The Log_10_ gc/mL reduction was obtained by comparing the results of suspensions inactivated at different conditions and assessed with two RT-qPCRs assays (A and B), with suspensions treated with different intercalating dyes. Abbreviations: gc, genome copies; <LoD, below limit of detection. Values in brackets indicate the number of positives replicates over the total number of replicates.

|  |  | Mean Log_10_ gc/mL | | | |  | Mean Log_10_ gc/mL reduction | | | |
| --- | --- | --- | --- | --- | --- | --- | --- | --- | --- | --- |
| Samples | **Treatment** | **Heat-Inactivated**  **70ºC for 1h** | | **Heat-Inactivated**  **99ºC for 5’** | |  | **Log_10_ gc/mL reduction**  **70ºC for 1h** | | **Log_10_ gc/mL reduction**  **99ºC for 5’** | |
|  |  | **assay A** | **assay B** | **assay A** | **assay B** |  | **assay A** | **assay B** | **assay A** | **assay B** |
| Pasteurized Milk  5-fold diluted | **Non-treated** | 5.65 ± 0.08  (4/4) | 5.36 ± 0.12  (4/4) | 5.63 ± 0.14  (4/4) | 5.26 ± 0.19  (4/4) |  | - |  |  |  |
|  | **PtCl_4_** | 4.53 ± 0.04  (4/4) | 4.00 ± 0.40  (4/4) | 2.79 ± 0.13  (3/4) | 3.07 ± 0.19  (4/4) |  | 1.12 ± 0.04 | 1.35 ± 0.27 | 2.84 ± 0.01 | 2.19 ± 0.37 |
|  | **PMAxx^TM^** | 5.45 ± 0.02  (4/4) | 5.32 ± 0.05  (4/4) | 5.28 ± 0.06  (4/4) | 4.85 ± 0.09  (4/4) |  | 0.20 ± 0.06 | 0.09 ± 0.07 | 0.35 ± 0.07 | 0.41 ± 0.28 |
|  | ***Crosslinker*** | 5.25 ± 0.02  (4/4) | 4.80 ± 0.00  (4/4) | 5.06 ± 0.29  (4/4) | 4.41 ± 0.18  (4/4) |  | 0.40 ± 0.11 | 0.56 ± 0.12 | 0.57 ± 0.16 | 0.86 ± 0.01 |
| Raw Milk  5-fold diluted | **Non-treated** | 5.57 ± 0.06  (4/4) | 5.10 ± 0.12  (4/4) | 5.30 ± 0.03  (4/4) | 4.87 ± 0.05  (4/4) |  | - |  |  |  |
|  | **PtCl_4_** | 2.60 ± 0.45  (3/4) | 2.58 ± 0.61  (2/4) | <LoD  (0/4) | <LoD  (0/4) |  | 2.97 ± 0.39 | 3.70 ± 1.55 | 4.59 ± 0.03 | 4.66 ± 0.05 |
|  | **PMAxx^TM^** | 5.15 ± 0.01  (4/4) | 4.72 ± 0.12  (4/4) | 4.89 ± 0.04  (4/4) | 4.38 ± 0.15  (4/4) |  | 0.42 ± 0.07 | 0.37 ± 0.00 | 0.41 ± 0.08 | 0.50 ± 0.20 |
|  | ***Crosslinker*** | 4.75 ± 0.12  (4/4) | 3.98 ± 0.20  (4/4) | 4.61 ± 0.18  (4/4) | 3.84 ± 0.06  (4/4) |  | 0.83 ± 0.06 | 1.12 ± 0.08 | 0.69 ± 0.21 | 1.04 ± 0.11 |

**Supplementary Table S9**. Mean recovery and coefficients of variation (CV) for HPAI H5N1 and PEDV, based on the sponge method (Method A), on artificially contaminated stainless steel and food-grade silicone (n=3) surfaces (10 cm^2^) using PBS and raw milk suspensions. Values between slashes indicate recovery ranges, and values between brackets indicate the ratio of positive replicates to the total number of replicates. Differents letters show statistically differences between virus mean recovery yields in each surface. Abbreviations: gc, genome copies; <LoD, below limit of detection.

| Sample | Surface | HPAI H5N1 | | | | | PEDV | | |
| --- | --- | --- | --- | --- | --- | --- | --- | --- | --- |
|  |  | **Assay A** | |  | **Assay B** | |  |  | |
|  |  | **Recovery** | **CV (%)** |  | **Recovery** | **CV (%)** |  | **Recovery** | **CV (%)** |
| PBS | **Stainless Steel** | 0.46 ± 0.11^a^  /0.36 – 0.60/  (4/6) | 24.23 |  | 0.10^a^  (1/6) | - |  | 6.54 ± 3.13^b^  /2.57 – 9.42/  (6/6) | 47.76 |
|  | **Food-grade silicone** | 2.12 ± 0.40^a^  /1.48 – 2.50/  (6/6) | 18.73 |  | 1.23^b^  (1/6) | - |  | 1.61 ± 0.32^a^  /1.28 – 2.01/  (6/6) | 20.05 |
| Raw Milk | **Stainless Steel** | <LoD^a^  (0/6) | - |  | <LoD^a^  (0/6) | - |  | 0.49 ± 0.15^b^  /0.29 – 0.67/  (6/6) | 31.23 |
|  | **Food-grade silicone** | <LoD^a^  (0/6) | - |  | <LoD^a^  (0/6) | - |  | 0.20 ± 0.08^b^  /0.14 – 0.33/  (5/6) | 40.74 |

**Supplementary Table S10**. Mean recovery and coefficients of variation (CV) for HPAI H5N1 and PEDV, based on the ISO 15216:1 method (Method B), on artificially contaminated stainless steel and food-grade silicone (n=3) surfaces (10 cm^2^) using PBS and raw milk suspensions. Values between slashes indicates recovery ranges in percentages, and values between brackets indicates the ratio of positive replicates to the total number of replicates. Abbreviations: gc, genome copies; <LoD, below limit of detection.

| Sample | Surface | HPAI H5N1 | | | |  | IAV H3N2 | |  | MgV | |  | PEDV | |
| --- | --- | --- | --- | --- | --- | --- | --- | --- | --- | --- | --- | --- | --- | --- |
|  |  | **RT-qPCR Assay A** | | **RT-qPCR Assay B** | |  |  |  |  |  |  |  |  |  |
|  |  | **Recovery** | **CV (%)** | **Recovery** | **CV**  **(%)** |  | **Recovery** | **CV**  **(%)** |  | **Recovery** | **CV**  **(%)** |  | **Recovery** | **CV**  **(%)** |
| PBS | **Stainless Steel** | 4.69 ± 3.91  /1.54 – 10.81/  (6/6) | 83.36 | 1.60 ± 1.05  /0.25 – 3.03/  (6/6) | 65.56 |  | 18.27 ± 3.32  /13.70 – 23.06/  (6/6) | 18.17 |  | 34.01 ± 8.03  /24.82 – 45.60/  (6/6) | 23.61 |  | 18.52 ± 7.55  /7.72 – 27.78/  (6/6) | 40.74 |
|  | **Food-grade silicone** | 26.86 ± 8.82  /15.43 – 37.30/  (6/6) | 32.86 | 14.72 ± 8.03  /10.03 – 30.97/  (6/6) | 54.54 |  | 11.87 ± 3.72  /6.66 – 15.86/  (6/6) | 31.36 |  | 21.46 ± 8.78  /9.65 – 29.26/  (6/6) | 40.93 |  | 29.35 ± 11.87  /18.63 – 45.08/  (6/6) | 40.43 |
| Raw Milk | **Stainless Steel** | 5.13 ± 2.34  /3.47 – 6.78/  (4/6) | 45.73 | 1.17 ± 0.59  /0.57 – 1.96/  (4/6) | 50.34 |  | 3.51 ± 0.97  /2.42 – 5.09/  (6/6) | 27.61 |  | 30.87 ± 10.78  /17.91 – 47.65/  (6/6) | 34.93 |  | 24.44 ± 5.46  /16.71 – 32.86/  (6/6) | 22.35 |
|  | **Food-grade silicone** | <LoD  (0/6) | - | <LoD  (0/6) | - |  | 5.07 ± 0.66  /4.45 – 6.15/  (6/6) | 13.46 |  | 31.73 ± 11.71  /16.81 – 47.09/  (6/6) | 36.92 |  | 25.52 ± 7.58  /16.45 – 34.33/  (6/6) | 29.70 |

**Supplementary Table S11**. P-values of the statistical analyses evaluating the effect of surface (stainless steel and food-grade silicone) and matrix (PBS and raw milk) on HPAI H5N1 and IAV H3N2 viral recovery. Abbreviations; NA, not analysed.

|  |  | **PBS** | | **Raw milk** | |  |
| --- | --- | --- | --- | --- | --- | --- |
|  |  | **Stainless Steel** | **Food-grade silicone** | **Stainless Steel** | **Food-grade silicone** | **Virus**  **(RT-qPCR Assay)** |
| **PBS** | **Stainless Steel** |  | 0.0002 | 0.1280 | **NA** | **HPAI H5N1 (A)** |
|  |  |  | 0.0017 | 1.0000 |  | **HPAI H5N1 (B)** |
|  |  |  | 0.0104 | 0.0022 |  | **IAV H3N2 (A)** |
|  | **Food-grade silicone** | 0.0002 |  | **NA** | 0.0028 | **HPAI H5N1 (A)** |
|  |  | 0.0017 |  |  | 0.0028 | **HPAI H5N1 (B)** |
|  |  | 0.0104 |  |  | 0.0051 | **IAV H3N2 (A)** |
| **Raw milk** | **Stainless Steel** | 0.1280 | **NA** |  | 0.0284 | **HPAI H5N1 (A)** |
|  |  | 1.0000 |  |  | 0.0284 | **HPAI H5N1 (B)** |
|  |  | 0.0022 |  |  | 0.0655 | **IAV H3N2 (A)** |
|  | **Food-grade silicone** | **NA** | 0.0028 | 0.0284 |  | **HPAI H5N1 (A)** |
|  |  |  | 0.0028 | 0.0284 |  | **HPAI H5N1 (B)** |
|  |  |  | 0.0051 | 0.0655 |  | **IAV H3N2 (A)** |
